# Work incentives, chronic illnesses and how sickness certificates are written affect sickness absence

**DOI:** 10.1080/02813432.2019.1571000

**Published:** 2019-02-20

**Authors:** Hans Thulesius

**Affiliations:** a Department of Clinical Sciences, Malmö, Family Medicine, Lund University; b Department of Research and Development, Region Kronoberg, Växjö, Sweden

To assess sickness absence and writing sickness certificates is in most countries the tasks of physicians in general and primary care physicians (PCPs) in particular. Sick leave, sickness absence and sick-listing are popular topics in our journal as seen in two papers in this issue by Shutzberg and by Vuorio et al. [1,2] as well as in many recently published articles [3–8].

At first it seems perfectly appropriate that PCPs should deal with sickness absence and sick-listing. But what if common disease concepts are inadequate to explain sick leave behavior since work capacity shows little correlation with disease severity? [9]. Then the PCP´s role in the sickness assessment process becomes less self evident.

 I and a physiotherapist researcher generated a grounded theory of *reincentivizing work* after researching sickness absence and sick-listing for some years by interviews, observations and literarure data [10]. Reincentivizing deals with work absence and how people are motivated or demotivated to work, typically in a welfare state context. Many reincentivizing variables have little to do with traditional health issues but instead with motivating and demotivating factors derived from all parts of human behavior, work environment and workplace conditions. *Reincentivizing* emphasises fellowship, identity, meaning, desire, plight, pride, and “flow” as motivational factors that are part of what we call *work drivers* or properties of different *behaviour modes*. Accordingly, work disability is defined as *hurt work drivers* and by people getting caught in *behaviour mode traps*. *Work drivers* are specified as work capacities + work incentives, monetary and non-monetary. Also, people can get trapped in certain *behavior modes* through changed capacities or incentives, or by inertia. Different modes have different drivers and these may trap the individual from *reincentivizing*, ie from going back to work or go on working. Driver assessments are done on several different levels. First by mode driver calculations by the worker. Work driver assessments are also done by employers, social insurance agency officials, physicians and eventually rehabilitation specialists including occupational health physicians, who can be mediators between the worker and the employer.

In this issue of the SJPHC Schutzberg found that Swedish physicians’ assessments of sickness were adapted to fit gatekeeping requirements of the social insurance agency in order to grant the patient sick leave [1]. Thus the physicians made it easier for patients to attain sick leave by using unsanctioned techniques to improve the chance of the sickness certificate to be accepted by the social insurance agency. These unsanctioned techniques involved exaggerations, omissions, adaptations and buzzwords that were thus disincentivising work. The Swedish social insurance agency’s gatekeeping activities is a *reincentivizing* property we call *controlling sick leave insurance* [10].

To reincentivize work is either done by repair strategies for hurt *work drivers* with *body repair* belonging to the health care domain in the case of an injury, a stroke, cancer treatment or an exacerbation of a chronic illness. Chronic conditions were in the study by Vuorio et al in this issue of the SJPHC found to have a stronger correlation to sickness absence than any other factor with a linear relationship between sickness absence days and the number of chronic diseases up to 5–6 diseases [2]. The correlation between chronic disease and sickness absence thus tells us that PCPs still have an important role to play in assessing sickness absence and writing sickness certificates!
